# Shengmai San Ameliorates Myocardial Dysfunction and Fibrosis in Diabetic* db/db* Mice

**DOI:** 10.1155/2016/4621235

**Published:** 2016-04-21

**Authors:** Juan Zhao, Tong-Tong Cao, Jing Tian, Hui-hua Chen, Chen Zhang, Hong-Chang Wei, Wei Guo, Rong Lu

**Affiliations:** Department of Pathology, Shanghai University of Traditional Chinese Medicine, 1200 Cailun Road, Shanghai 201203, China

## Abstract

In this study, we mainly investigated the effects of Shengmai San (SMS) on diabetic cardiomyopathy (DCM) in* db/db* mice. The* db/db* mice were randomly divided into model group and SMS group, while C57BLKS/J inbred mice were used as controls. After 24-week treatment, blood glucose, body weight, and heart weight were determined. Hemodynamic changes in the left ventricle were measured using catheterization. The myocardial structure and subcellular structural changes were observed by HE staining and electron microscopy; the myocardium collagen content was quantified by Masson staining. To further explore the protective mechanism of SMS, we analyzed the expression profiles of fibrotic related proteins. Compared to nondiabetic mice,* db/db* mice exhibited enhanced diastolic myocardial dysfunction and adverse structural remodeling. Higher expression of profibrotic proteins and lower levels of extracellular matrix degradation were also observed. After SMS oral administration for 24 weeks, cardiac dysfunction, hypertrophy, and fibrosis in diabetic mice were greatly improved. Moreover, increased profibrotic protein expression was strongly reversed by SMS treatment in* db/db* mice. The results demonstrate that SMS exerts a cardioprotective effect against DCM by attenuating myocardial hypertrophy and fibrosis via a TGF-*β* dependent pathway.

## 1. Introduction

Diabetic patients have a 2- to 5-fold increased risk of developing heart failure [1], which is partly driven by diabetic cardiomyopathy (DCM). DCM is characterized by diastolic dysfunction, myocardial fibrosis, and hypertrophy without ischemic heart disease, hypertension, or other comorbidities. The pathogenesis of DCM is complicated, but myocardial fibrosis, which increases ventricle stiffness, is thought to be one of the major causes of myocardial dysfunction [2]. Numerous studies demonstrated that one of the key determinants of myocardial fibrosis is the accumulation of increased extracellular matrix (ECM), which causes irreversible tissue damage and consequent cardiac dysfunction, ultimately resulting in heart failure [3]. Among the cytokines in the regulation of ECM metabolism, transforming growth factor-*β*1 (TGF-*β*1) is the critical factor and has been recognized as a therapeutic target for organ fibrosis [4].

*Shengmai San* (SMS), a traditional Chinese medical recipe, consists of Radix Ginseng (*Panax ginseng*, Araliaceae), Radix Ophiopogonis (*Ophiopogon japonicas*, Liliaceae), and Fructus Schisandrae (*Schisandra chinensis*, Schisandraceae). SMS is deemed a typical replenishing Qi and nourishing Yin formula in clinic and has been traditionally used in ischemic disease and diabetic patients. In experimental studies, SMS has been reported to have multiple pharmacological activities, such as antioxidant and anti-inflammation activities and being a regulator of lipid metabolism [5, 6]. Recently, one study indicated that SMS exhibited an antimyocardial fibrosis effect in a rat model induced by high-fat diet and STZ injection [7]; however, the exact mechanism remains to be determined. Thus, in the present study, we investigated the effects of SMS on cardiac function and fibrosis in a type 2 diabetic* db/db* mouse model, to further observe the associated signaling mechanism.

## 2. Materials and Methods

### 2.1. Shengmai San Preparation

*Shengmai San* (SMS) was purchased from Tauto Biotech Company (Shanghai, China), which contains ginseng 10 g, ophiopogon root 15 g, and Schisandra chinensis 6 g, and the solution was concentrated to 0.8 g/mL.

### 2.2. Animals and Treatment

Experimental protocols complied with the National Institutes of Health Guidelines on the Use of Laboratory Animals and were approved by the Animal Care and Use Committee of Shanghai University of Traditional Chinese Medicine. Thirty male BKS·Cg-m+/+LeprdbNJU mice (*db/db* mice), 4–6 weeks age, were randomly divided into two groups: model group and SMS group. Fifteen male C57BL/6J mice were used as age-matched controls. All animals were purchased from the Model Animal Research Center of Nanjing University (Nanjing, China) and raised in the animal research institute of Shanghai University of TCM (Shanghai, China). The mice were kept on a 12 h light/dark cycle and had access to food and water ad libitum. Since 8 weeks age, the mice were treated with SMS at a dose of 4.5 g/kg daily or with same volume of vehicle by oral gavage for 24 weeks.

### 2.3. Hemodynamic Measurement

At the end of the experiment, mice were fasted for 12 hours and blood samples were collected from the tail vein. Blood glucose levels were tested using a digital blood glucose meter (Optium Xceed, Abbott Laboratories, USA). Body weight and heart weight of mice were measured before or after hemodynamic measurements, respectively. Cardiac function was determined by invasive hemodynamic measurements. Mice were anesthetized with pentobarbital sodium (60 mg/kg, i.p.). With the help of stereoscopic microscope (Stemi DV4, Carl Zeiss, Germany), a SciSence FT-1.2 catheter (Scisence, Canada) was inserted into the right carotid artery and advanced into the left ventricle. The ventricular pressure was recorded and analyzed with Labscribe 2 software (iWorx, Dover, NH, USA). The hemodynamic parameters included maximal ascending and descending rates of left ventricular pressure (+*dp*/*dt*_max_ and −*dp*/*dt*_max_), left ventricular systolic pressure (LVSP), and left ventricular end-diastolic pressure (LVEDP).

### 2.4. Hematoxylin and Eosin (H&E) Staining

After hemodynamic measurements, mouse hearts were excised from the chest, trimmed of atria and large vessels, weighed, and transversely cut between the atrioventricular groove and the apex. The specimens were fixed in 4% paraformaldehyde. The tissues were paraffin-embedded and sliced perpendicular to interventricular septum continuously. The sections underwent hematoxylin and eosin staining and then were investigated by optical microscope (Stemi DV4 or Axio Scope A1, Carl Zeiss, Germany).

### 2.5. Myocardial Ultrastructure Observation

Parts of cardiac tissues were rapidly cut into 1 mm cubes, immersed in 2.5% glutaraldelyde in 0.1 M phosphate buffer (pH 7.4) overnight at 4°C. After fixation, the selections were immersed in 1% buffered osmium tetroxide for 2 h. The specimens were then dehydrated through a graded ethanol series and embedded in epoxy resin. After that, the selections were incised into ultrathin sections (60–70 nm) with an ultramicrotome and poststained with uranyl acetate and lead citrate. Then sections were examined under a Tecnai-12 Biotwin transmission electron microscope (Philips, Germany).

### 2.6. Masson's Trichrome Staining and Collagen Volume Fraction (CVF) Analysis

After conventional deparaffin of the paraffin sections, Masson's trichrome staining was performed to evaluate myocardial fibrosis. Myocardial cells were stained red and collagenous fibers stained blue. The collagen deposition was quantitatively analyzed for collagen volume fraction (CVF) via Metamorph image process (Universal Imaging Corp, USA). The calculation formula of CVF in each view of the slice is CVF = collagen area/total area × 100%.

### 2.7. Western Blotting

Total extracted protein lysates from similar portions of LV tissue were prepared by standard procedures, and protein lysate concentrations were determined via BCA protein assay kit (Beyotime, Shanghai, China). Equivalent amounts of tissue protein (50 *μ*g) were subjected to 10–12% SDS-PAGE, electrotransferred onto polyvinylidene difluoride (PVDF) membranes, and then incubated overnight at 4°C with the primary antibodies, including transforming growth factor-*β*1 (TGF-*β*1), transforming growth factor-*β* receptor II (TGF*β*RII), p-Smad2, p-Smad3, Smad2, Smad3, Smad4 (Cell Signaling Technology, USA), matrix metallopeptidase-2 (MMP-2), matrix metallopeptidase-9 (MMP-9), tissue inhibitor of metalloproteinases-2 (TIMP-2), and GAPDH (Santa Cruz biotechnology, USA). Next day, membranes were washed and incubated with the corresponding secondary antibodies for 2 h; anti-mouse and anti-rabbit antibodies were from Cell Signaling Technology; protein bands were detected by Fluor Chem E (Protein Simple, USA). Quantitation was performed via Image J software (Bethesda, MD, USA). GAPDH was used as loading controls for total protein expression.

### 2.8. Statistical Analysis

All values were analyzed with SPSS21.0 and expressed as means ± SEM. Multiple comparisons between groups were examined using one-way analysis of variance (ANOVA) followed by Tukey's post hoc analysis, and *P* < 0.05 were considered statistically significant.

## 3. Results

### 3.1. Effects of SMS on Blood Glucose Level, Body Weight, and Heart Weight

**Figure 1:**
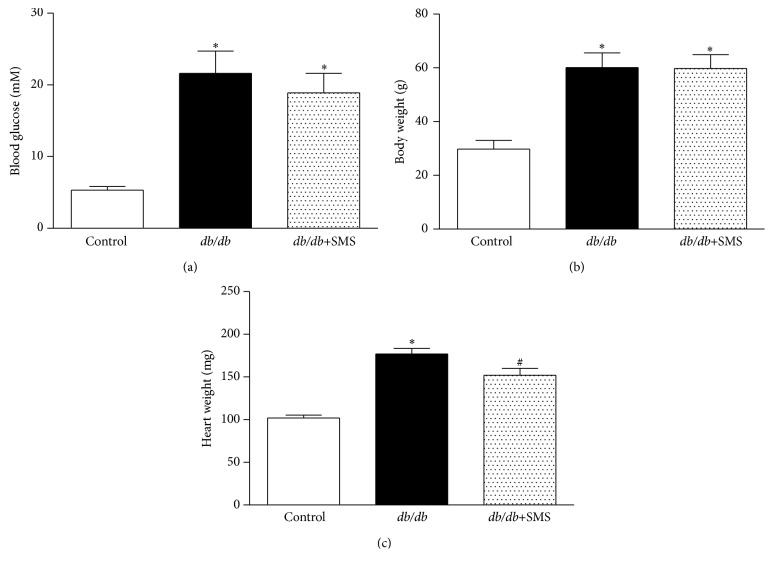
Effects of SMS on blood glucose, body weight, and heart weight. After a 24-week treatment, general biochemical parameters were measured (*n* = 8–10). In the bar figures, values are the mean ± SEM. ^*∗*^ *P* < 0.05 versus Control group; ^#^ *P* < 0.05 versus* db/db* group. (a) Fast blood glucose level, (b) body weight, and (c) heart weight.

Compared to nondiabetic controlmice,* db/db* mice exhibit typical characteristics of type 2 diabetes indicated by hyperglycemia as well as increasing heart and body weight. Blood glucose levels and body weight did not significantly differ between* db/db* mice with or without SMS treatment; however, SMS markedly alleviated the increase of heart weight seen in* db/db* mice (Figure 1).

### 3.2. SMS Attenuated Diabetes-Induced Myocardial Diastolic Dysfunction

**Figure 2:**
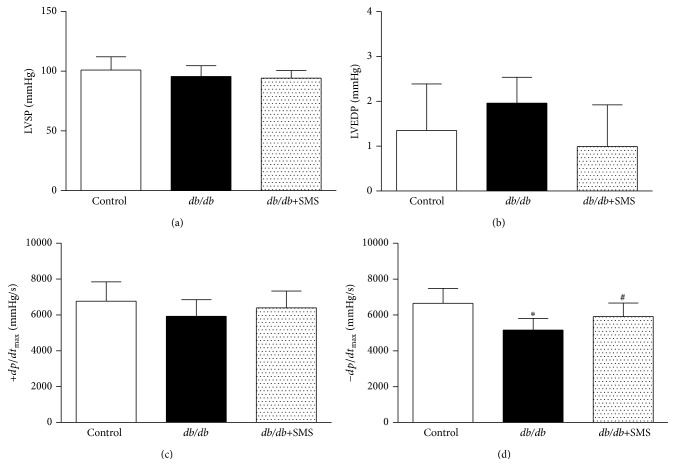
Effects of SMS on diabetes-induced myocardial dysfunction. Left ventricular function was assessed by cardiac catheterization in anesthetized mice (*n* = 8–10). In the bar figures, values are the mean ± SEM. ^*∗*^ *P* < 0.05 versus Control group; ^#^ *P* < 0.05 versus* db/db* group. (a) Left ventricular systolic pressure (LVSP), (b) left ventricular end-diastolic pressure (LVEDP), (c) maximal ascending rates of left ventricular pressure (+*dp*/*dt*_max_), and (d) maximal descending rates of left ventricular pressure (−*dp*/*dt*_max_).

To determine the role of SMS in type 2 diabetic mice, cardiac catheterization was performed to evaluate ventricular function. The results demonstrated that LVSP and +*dp*/*dt*_max_ values did not significantly differ between nondiabetic and* db/db* mice, suggesting unaltered systolic function in type 2 DM (Figures 2(a) and 2(c)). In contrast, compared with the control, −*dp*/*dt*_max_ value was significantly reduced in* db/db* mice, accompanied with increased LVEDP level, indicating a marked diastolic dysfunction in* db/db* mice, although increased LVEDP in* db/db* mice did not show statistical significance. Moreover, SMS treatment for 24 weeks significantly reversed the diastolic dysfunction in* db/db* mice (Figures 2(b) and 2(d)).

### 3.3. Effects of SMS on Histological and Morphological Changes

**Figure 3:**
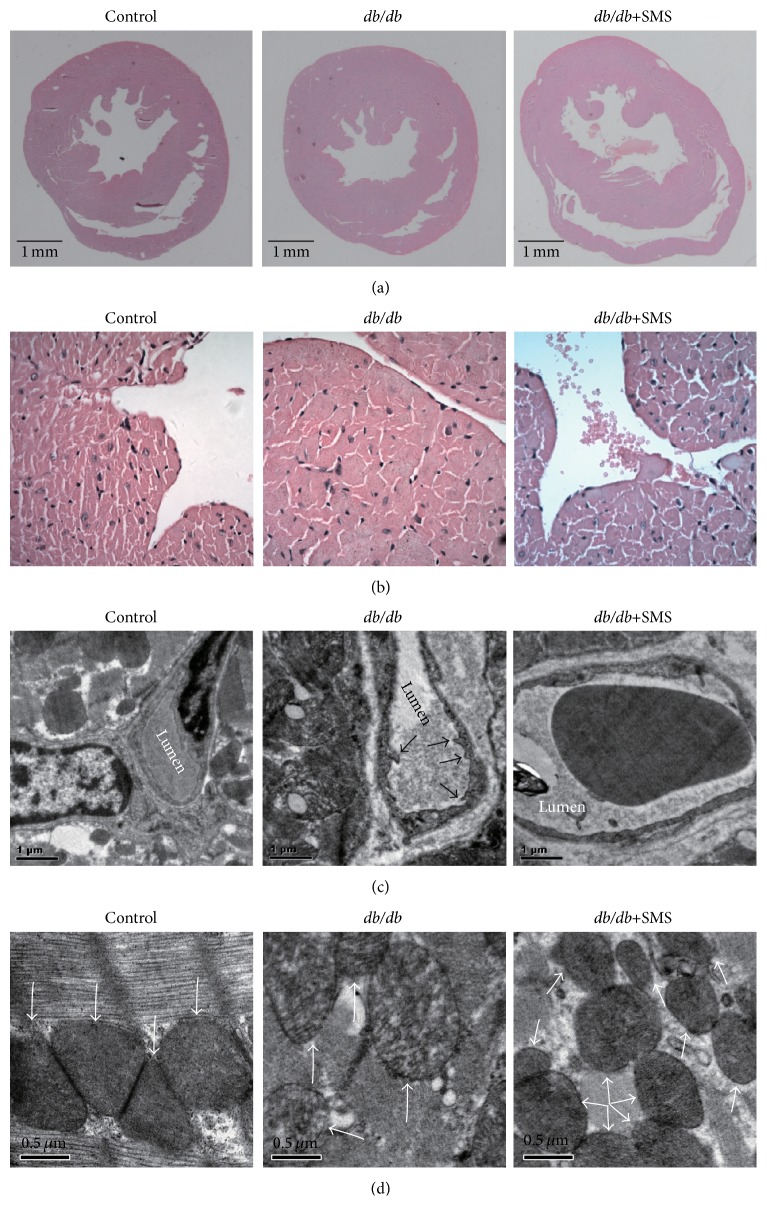
Effects of SMS on histological and ultrastructure changes. To assess histological and morphological features in each group, H&E staining and transmission electron microscopy (TEM) observation were performed. (a) Low-power (8-fold) and (b) high-power (200-fold) via light microscope; (c) and (d) myocardial ultrastructure images from TEM. (Black arrows indicate finger-like projections, while white arrows indicate mitochondria.)

To identify the morphological changes in the* db/db* mice and the protective effects of SMS, heart sections were processed for HE staining and TEM. HE images of the model group displayed remarkably thickened ventricular wall, hypertrophied cardiomyocyte, and disordered cell arrangement. These responses were partly prevented by SMS (Figures 3(a) and 3(b)). TEM images of* db/db* hearts displayed small finger-like projections on the surface of endothelial cell (Figure 3(c), black arrows) and swollen mitochondria (Figure 3(d), white arrows), which were partly attenuated by SMS treatment.

### 3.4. Effects of SMS on Myocardial Fibrosis and TGF-*β*1 Expression

**Figure 4:**
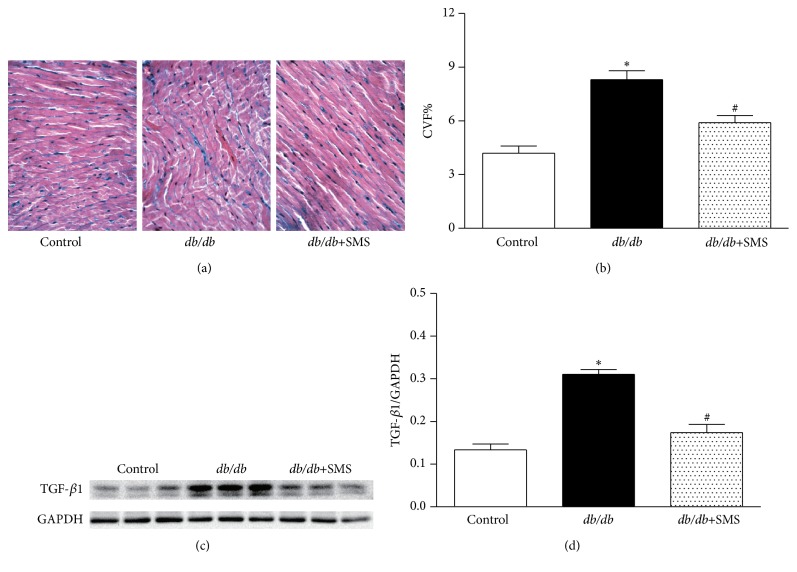
Effects of SMS on myocardial fibrosis and TGF-*β*1 expression. To detect the role of SMS in myocardial remodeling, Masson's trichrome staining was performed to evaluate fibrosis, the myocardial cells were stained for red, and collagenous fibers were blue. The collagen deposition was quantitatively analyzed via CVF, which equals collagen area/total area × 100% (a and b). To observe the underlying mechanism involved in antifibrosis of SMS, the level of transforming growth factor-*β*1 (TGF-*β*1) protein expression in heart tissue was determined using Western blotting (3 heart tissues from each group) (c and d). Data are presented as mean ± SEM. ^*∗*^ *P* < 0.05 versus Control group; ^#^ *P* < 0.05 versus* db/db* group.

Using Masson staining and CVF quantifiable analysis, we found that* db/db* mice exhibited more severe myocardial fibrosis compared to nondiabetic mice, indicated by increased blue collagenous fibers and higher CVF values. SMS administration for 24 weeks partly ameliorated cardiac hypertrophy and markedly reversed myocardial fibrosis in* db/db* mice (Figures 4(a) and 4(b)). To observe the underlying mechanism involved in antifibrosis of SMS, the level of TGF-*β*1 protein expression in heart tissue was determined by Western blotting. The data indicated that TGF-*β*1 was significantly higher in cardiac tissues of* db/db* mice compared tonondiabetic mice, suggesting activation of fibrosis associated pathway in diabetic mice. SMS administration significantly decreased the expression level of TGF-*β*1 protein in* db/db* mice (Figures 4(c) and 4(d)).

### 3.5. Effects of SMS on TGF-*β* Associated Downstream Signaling

**Figure 5:**
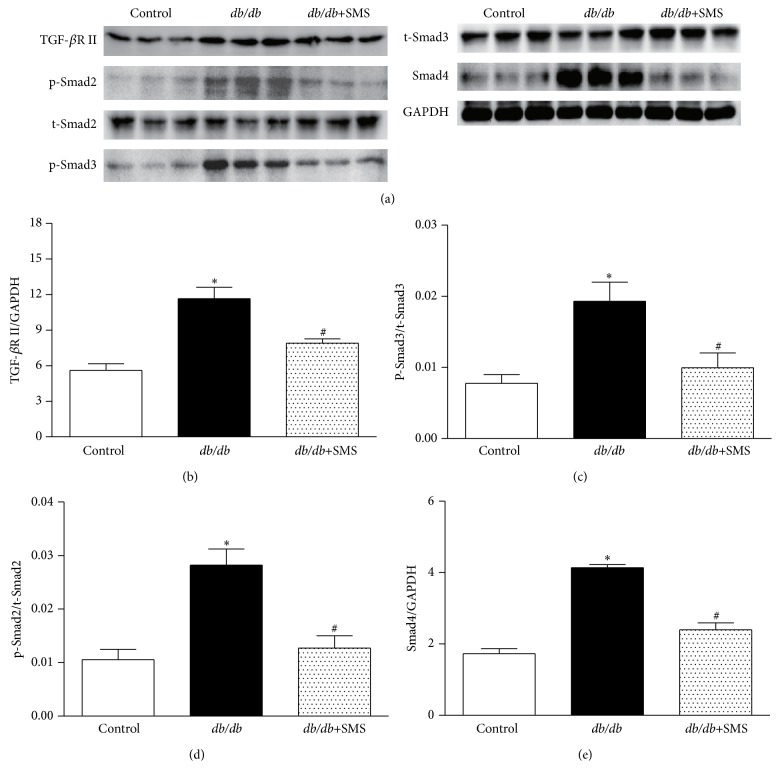
Effects of SMS on TGF-*β* associated downstream signaling. Protein expression was determined in the whole cell lysate via Western blotting and quantified by densitometric analysis. (a) Protein expression band. (b–e) Quantified value of protein expression. Data presented are means ± SEM (3 heart tissues from each group). ^*∗*^ *P* < 0.05 versus Control group; ^#^ *P* < 0.05 versus* db/db* group. TGF-*β*1, transforming growth factor-*β*1; TGF*β*RII, transforming growth factor-*β* receptor II; p-Smad2, phosphorylated Smad2; p-Smad3, phosphorylated Smad3; t-Smad2, total Smad2; t-Smad3, total Smad3; MMP-2, matrix metallopeptidase 2; MMP-9, matrix metallopeptidase 9; TIMP-2, tissue inhibitor of metalloproteinases-2.

As a critical factor for development of organ fibrosis, the expression of TGF-*β*1 dramatically increased in* db/db* mice. Based on the above result, we further analyzed the changes of TGF-*β* associated downstream signaling. The data shows that the levels of TGF-*β* receptor II, phospho-Smad2, phospho-Smad3, and Smad4 were significantly higher in cardiac tissues of* db/db* mice compared to nondiabeticmice, which was inhibited by SMS treatment for 24 weeks (Figure 5). No significant changes in total Smad2 or Smad3 protein levels were observed.

### 3.6. Effects of SMS on MMP-2, MMP-9, and TIMP-2 Levels

**Figure 6:**
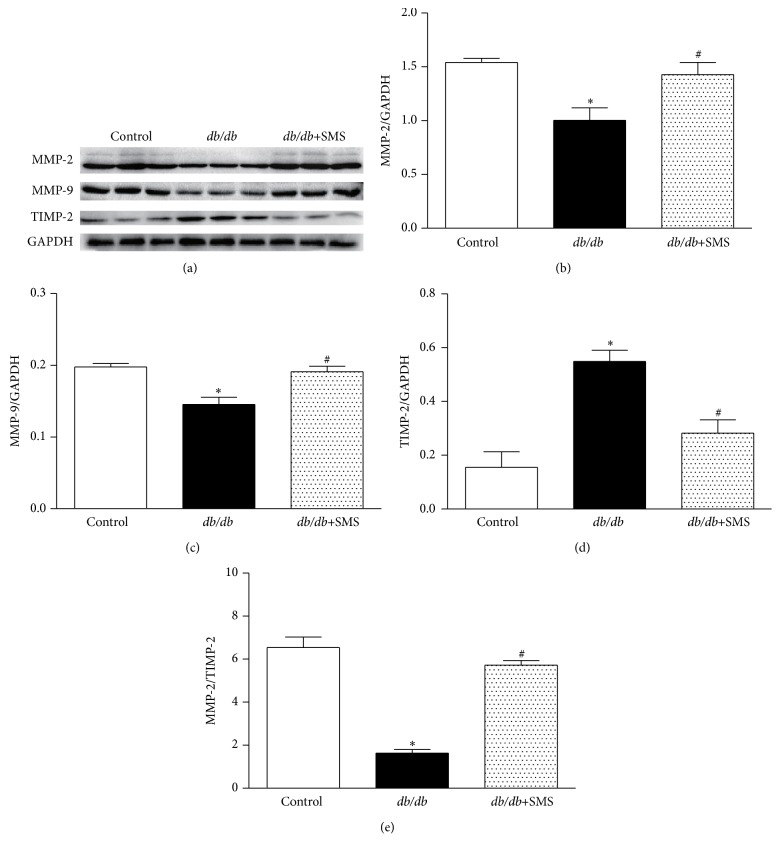
Effects of SMS on MMP-2, MMP-9, and TIMP-2 Levels. Protein expression was determined in the whole cell lysate via Western blotting and quantified by densitometric analysis. (a) Protein expression band. (b–e) Quantified value of protein expression. Data presented are means ± SEM (3 heart tissues from each group). ^*∗*^ *P* < 0.05 versus Control group; ^#^ *P* < 0.05 versus* db/db* group. MMP-2, matrix metallopeptidase 2; MMP-9, matrix metallopeptidase 9; TIMP-2, tissue inhibitor of metalloproteinases-2.

Myocardial ECM is primarily mediated by MMPs and their role in fibrosis is now well established; thus, we further assessed the expression of MMP-2, MMP-9, and TIMP-2. The data shows that MMP-2 and MMP-9 protein levels in* db/db* mice were significantly downregulated, whereas TIMP-2 levels were significantly upregulated compared to nondiabetic mice, leading to a lower MMP-2/TIMP-2 ratio in* db/db* mice. The changes of protein levels presented in diabetic mice were markedly reversed by SMS treatment (Figure 6).

## 4. Discussion

In spite of the clinical importance of diabetic cardiomyopathy (DCM) as a distinct disease, the cellular and molecular mechanisms triggering the adverse changes in diabetic myocardium have not been fully understood. In our study, compared with nondiabetic control mice, the 32-week* db/db* mice exhibited obvious obesity, hyperglycemia, myocardial hypertrophy, and fibrosis. Using hemodynamic analysis, we also found distinct diastolic dysfunction in* db/db* mice, indicated by a significant decreased −*dp*/*dt*_max_ and an elevated LVEDP, although increased LVEDP did not reach statistical significance. In contrast, myocardial systolic function in* db/db* mice was similar tonondiabetic mice, characterized by unaltered LVSP and +*dp*/*dt*_max_. In type 2 DM experimental models, cardiac performance has been extensively studied. As a leptin receptor defective animal, most studies support that* db/db* mice mainly display diastolic dysfunction, while systolic function was preserved well for a long time [8, 9], which was also confirmed by our results. Since myocardial hypertrophy and fibrosis cause increased passive myocardial stiffness, we assumed that the two features, at least in part, result in diastolic dysfunction in* db/db* mice.

SMS treatment has been proved effective for type 2 diabetes in both clinical and experimental studies. Some clinical researches indicated that SMS promotes the beneficial effects of oral hypoglycemic drugs and improves diabetic complications [10]. In experimental studies, multiple pharmacological activities of SMS have also been reported, such as antioxidant, anti-inflammation, and regulating lipid metabolism activities. In our study, SMS treatment for 24 weeks significantly reversed the myocardial diastolic dysfunction shown in* db/db* mice. Furthermore, SMS apparently prevented cardiac hypertrophy and ultrastructural injury, which was confirmed by histological and morphological assessment. We found that heart weight was significantly increased in* db/db* mice, with markedly enlarged cardiomyocyte cross-sectional area, accompanied by swollen mitochondria, all of which were alleviated by SMS administration. Masson staining and CVF analysis revealed that SMS also reversed the increased myocardial fibrosis shown in* db/db* hearts. It is noteworthy that blood glucose and body weight did not significantly differ between* db/db* and* db/db* treated with SMS, suggesting that hypoglycemic effect may not be involved. In conclusion, our data indicated that SMS attenuate diastolic dysfunction, cardiac hypertrophy, and fibrosis in type 2 diabetic* db/db* mice model.

Fibrotic remodeling of the myocardium has been reported to play a critical role in pathophysiologic progress in DCM [11, 12]. The potential causes of myocardial fibrotic remodeling include the imbalance between extracellular matrix synthesis and degradation and interstitial collagen deposition and disorder of collagen proportions, which gradually lead to myocardial stiffness and eventually cardiac dysfunction [13–16]. Our data shows that SMS remarkably abrogated the accumulation of collagen. Consistent with our results, recent evidence implicates that SMS contribute to antimyocardial fibrosis in a rat model induced by high-fat diet and STZ injection [7]. However, the exact mechanism of antifibrosis of SMS is still unclear.

Among numerous fibrotic signals, TGF-*β*1 is reported to be a key fibrogenic mediator. Excessive activation of TGF-*β*1 leads to dysfunction of extracellular matrix synthesis and degradation, which results in fibrotic remodeling [17, 18]. It is now clear that the binding of TGF-*β*1 to its receptors promotes Smad2 and Smad3 phosphorylation, after forming heterotrimers with co-Smad (Smad4), then translocating into the nucleus to regulate gene transcription. Smad2 and Smad3 are well-documented downstream mediators of TGF-*β*1 induced fibrosis, and activation of Smad2 and Smad3 is found to stimulate matrix-component synthesis, such as fibronectin (Fn), collagens, and proteoglycan [19–22]. Moreover, TGF-*β*1 can also inhibit the expressions of MMPs, which are the main degrading enzymes of the ECM, by increasing plasminogen activator inhibitors (PAI) and decreasing plasminogen activator (PA), and mediate the synthesis of protein hydrolytic enzyme inhibitors [23–26]. In the present study, we found that as a critical factor for development of organ fibrosis, the expression of TGF-*β*1 and TGF-*β* receptor II dramatically increased in* db/db* mice. We further detected changes in TGF-*β* associated downstream signaling; the data showed that the levels of p-Smad2, p-Smad3, and Smad4 were significantly higher in cardiac tissues of* db/db* mice compared to nondiabeticmice, without significant changes in total Smad2 and Smad3 protein; all changes in protein levels were reversed by SMS treatment. Furthermore, MMP-2 and MMP-9 in* db/db* mice were markedly downregulated, accompanied by higher level of TIMP-2 than nondiabetic mice, which led to a much lower ratio of MMP-2/TIMP-2 in* db/db* mice. After SMS administration, the ratio of MMP-2/TIMP-2 nearly shifted to normal condition. The above data implies that an imbalance of extracellular matrix synthesis and degradation may aggravate fibrotic remodeling in* db/db* mice, which consequently causes cardiac dysfunction.

Taken together, our results demonstrate that as a traditional Chinese medical recipe, SMS exerts a protective effect against type 2 diabetes-induced myocardial dysfunction and fibrosis through the regulation of TGF-*β*1/Smads axis. Therefore, SMS should be a potential drug for the treatment of diabetic cardiomyopathy.
